# Advancing infantile hemangioma diagnosis by integrating temperature, color, and texture

**DOI:** 10.1117/1.JBO.30.7.075001

**Published:** 2025-07-17

**Authors:** José Antonio Pérez-Carrasco, Carmen Serrano, Juan Antonio Leñero-Bardallo, José Bernabeu-Wittel, Begoña Acha

**Affiliations:** a Universidad de Sevilla, Department of Signal Theory and Communications, Sevilla, Spain; b Universidad de Sevilla, Department of Electronics and Electromagnetism, Sevilla, Spain; c Virgen de Rocío University Hospital, Dermatology Unit, Sevilla, Spain

**Keywords:** infantile hemangioma, texture, color, classification

## Abstract

**Significance:**

Infantile hemangiomas are one of the most prevalent benign tumors in childhood. Typically, diagnosis relies on visual assessment of area, texture, and color. A few studies have focused on various color attributes in superficial and mixed Infantile hemangioma types, neglecting the deep category. Limited research has explored temperature in the location of hemangioma lesions.

**Aim:**

We seek, for the first time, to quickly identify and classify infantile hemangioma lesions using a portable, programmable handheld device. The system aims to (1) replicate a physician’s assessment of infantile hemangioma and (2) deliver an easy way to understand automatic diagnosis.

**Approach:**

The custom-built device comprises an infrared sensor and a visible light spectrum sensor to assess color and depth through computations of different color and texture features. Over a 3-year period, 53 patients were monitored, and 83 hemangioma images were captured.

**Results:**

The device accurately localized all lesions in real time and classified hemangioma lesions into three primary types using selected color and texture features. Evaluation metrics showed an average sensitivity of 0.8948 and specificity of 0.7313 for an accuracy of 0.7572 and an average sensitivity of 0.7803 and specificity of 0.8720 for an F-score of 0.7826 in the three-class classification.

**Conclusions:**

The two-sensor device accurately identifies and categorizes infantile hemangioma lesions, providing a clear automated diagnosis based on computerized features.

## 1 Introduction

**Fig. 1:**
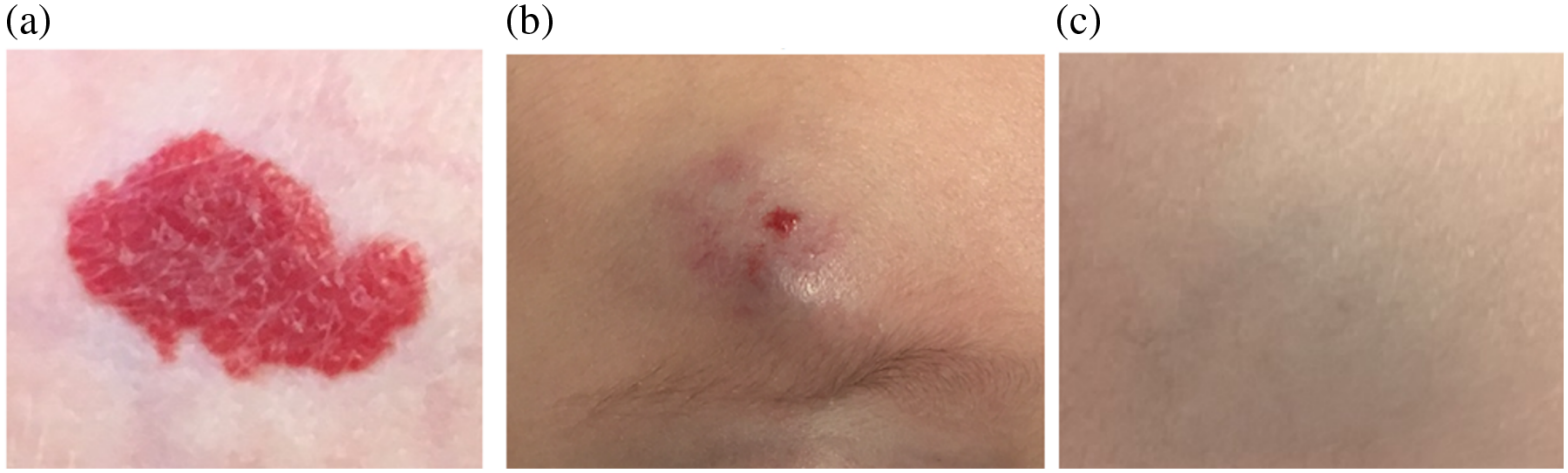
Different types of IH analyzed throughout this work: (a) superficial IH, (b) mixed IH, and (c) deep IH.

Infantile hemangiomas (IH) represent the most common benign tumors seen in childhood. They are estimated to affect ∼1% to 3% of all newborns, with the prevalence rising to around 10% in infants under 1 year of age.^1^^,^^2^ The diagnosis of the vast majority of IH cases is primarily based on clinical evaluation,^1^^–^^4^ where healthcare providers rely on visual inspection and palpation to assess key features such as size, texture, color, and depth for predicting clinical outcomes and guiding treatment decisions. Based on their color and depth, infantile hemangiomas can be categorized into superficial, mixed, or deep types (see Fig. 1).

**Fig. 2:**
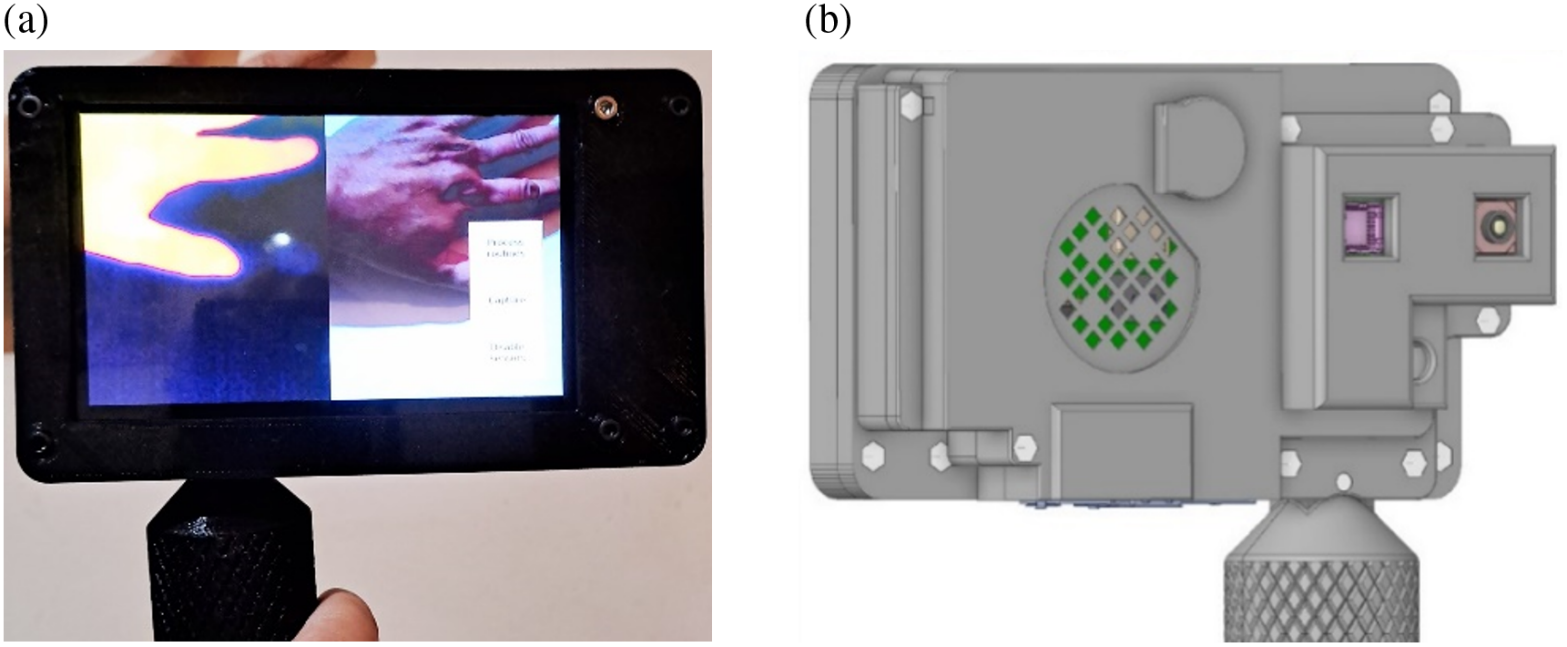
Front (a) and rear (b) views of the multimodal handheld device.

To assist doctors in diagnosing and treating IH effectively, the development of a computer-aided diagnosis tool capable of (1) mimicking a physician’s evaluation of IH by extracting color and texture data to estimate color and depth and (2) providing an understandable automatic diagnosis would be extremely attractive to clinicians in their day-to-day practice. The multimodal handheld device built by the authors and described in this study offers, for the first time, these two essential features. The device has two sensors, one in the infrared (IR) spectrum and the other in the visible spectrum. Figure 2 shows the front and rear views of the multimodal handheld device.

The use of the IR sensor, previously utilized by the authors,^5^^,^^6^ enables the localization and monitoring of IH lesions through the analysis of local temperature values. Expanding this approach, we use the second sensor to incorporate color and texture features to estimate lesion color and depth, thus enabling, for the first time, the classification of lesions into the three types of IH considered. The features are selected to closely match physicians’ visual examinations of lesion color and depth. Therefore, the system enhances the clinical visual diagnostic capabilities for IH lesions through the two sensors, providing daily clinical support by mimicking the clinician’s perceptual diagnostic approach.

In recent years, temperature-based techniques have emerged as valuable tools^5^^–^^10^ in aiding the diagnosis and precise localization of IH lesions, especially for deep IH lesions, which might not be visually apparent on the surface of the patient’s skin.

Only a few studies have given limited attention to the use of color and texture features in analyzing IH lesions,^11^^–^^20^ primarily focusing on the localization and segmentation of superficial and mixed hemangiomas due to their prevalence and simpler color analysis, and neglecting depth estimates and the possible presence of deep subcutaneous components.

Several notable works have delved into this area. Mohamed and Rabie^21^ presented an extensive review about artificial intelligence (AI) and digital imaging methods for detection, segmentation, and assessing the treatment response in IH cases. The study by Abagge et al.^14^ examined the evolution of IH color and size without delving into a depth assessment. This study, which involved 17 patients under 10 months, utilized Adobe Photoshop® software and a grading scale to assess lesion color. In another study by O’Brien et al.,^15^ colorimetric features were employed to differentiate between IH and port wine birthmarks in infants under 3 months, utilizing electronic colorimeters. However, reliance on such expensive additional hardware might limit widespread adoption. Sultana et al.^16^ have also contributed to this field with research focusing on the evolution of hemangiomas based on color and area measurements, and in a separate study,^17^ they explored the application of deep learning techniques for lesion segmentation, though not for classification purposes. Other works based on deep learning techniques were aimed at distinguishing lesions between IH and non-IH^22^^,^^23^ or classifying vascular anomalies.^24^^–^^26^ Zhang et al.^27^ compared different deep learning architectures for classification between hemangiomas and vascular malformations. In addition, in the work by Xie et al.,^28^ the authors analyzed red, green, and blue (RGB) values of superficial IHs before and after treatment to quantify changes in their colors.

Despite the importance of considering depth in hemangioma analysis, the integration of temperature data alongside color and texture features to enhance the localization, diagnosis, and monitoring of hemangiomas remains an underexplored area. Burkes et al.^29^ have made strides in the integration of temperature data alongside color and texture features to include depth in hemangioma analysis. They employed a combination of a Nikon camera, an Artec MHT scanner, and a FLIR T400 camera to analyze temperature and color features to study the evolution of such lesions. Their study was carried out with 25 subjects under 19 months old. However, the complexity and costliness of the equipment posed challenges for broader adoption in clinical settings. In addition, real-time results were not achievable with their current setup, highlighting the need for more accessible and practical approaches for comprehensive hemangioma assessment.

To the best of our knowledge, no published work has aimed to classify IH lesions into their three main subtypes: superficial, mixed, and deep. Moreover, there are no published works incorporating temperature information for the location and classification of IH lesions into those three subtypes. This study presents a cost-effective handheld device that accurately identifies and categorizes infantile hemangioma lesions, providing an automatic diagnosis based on computerized features correlated with clinical features.

## 2 Materials and Methods

### 2.1 Material

#### 2.1.1 Subjects

Fifty-three patients under 4 years old, each with at least one IH, were examined using the specified device during their initial dermatology appointment. This study yielded a total of 83 images: 44 depicting superficial IH, 27 showing mixed IH, and 12 illustrating deep IH. The database contains only one image of each lesion. This helps ensure that the same lesion is never included in both the training and test sets. Certain patients had multiple IH lesions. All clinical validations and on-site experiments were conducted over 3 years by two clinical experts and two technicians. The patients were monitored as the clinical experts simultaneously utilized conventional diagnostic methods (visual inspection, Doppler, and/or MRI as needed) alongside our custom device for the diagnosis and management of IH lesions. Each patient and lesion were subjected to a specific protocol where two distinct images (clinical and infrared) were captured simultaneously, maintaining a fixed distance and consistent lighting conditions. This study was performed in line with the principles of the Declaration of Helsinki. The Virgen del Rocío University Hospital Review Board granted ethics approval. Informed consent was obtained from the legal representatives of all subjects involved in the study.

#### 2.1.2 Image acquisition

The device comprises two image sensors (for infrared and visible light), a touch screen, a Raspberry Pi IV processor, and a thermometer (see Fig. 2).^5^^,^^6^ The two sensors were perfectly aligned along the X-direction and separated by 15 mm. Although their fields of view are not identical, the proposed arrangement and the optics mounted on the sensors ensure that both sensors consistently capture the region of interest when a snapshot of a hemangioma is taken. Images were captured at a distance of ∼30  cm. At this distance, and considering the typical dimensions of a hemangioma, both sensors can reliably capture the region of interest, provided the lesion is centered within the system.

The infrared sensor functions as a thermal imaging camera, detecting radiation in the long-wave infrared (LWIR) band. The second sensor is a standard digital camera operating in the visible light spectrum. The device captures images simultaneously from both sensors and features a split screen with one window displaying the clinical image in the visible spectrum and the other the thermal image (Fig. 2).

Below is a detailed description of the components:

• A single-board computer (SBC). We chose a Raspberry Pi IV board to build the prototype. The SBC controls all the peripherals and sensors. It has the Raspbian Linux-based operating system and can run real-time custom image processing algorithms. A user interface was programmed to acquire, display, and process the images. The SBC also has Wi-Fi and ethernet connectivity, and an expansion bus is available to add extra peripherals or sensors if necessary.
• A visible spectrum camera Sony IMX708. It has an image sensor operating in the visible spectrum, with a resolution of 4608×2592  pixels. The selected version includes a wide-angle lens and autofocus capabilities. The sensor features a MIPI CSI-2 interface, making it compatible with the Raspberry Pi IV board, which manages all system peripherals. In addition, the sensor supports HD video, enabling real-time visualization through the system interface.
• A FLIR Lepton 3.5 160×120  pixel thermal camera operates in the LWIR band. It is an infrared sensor, which is optimal for lower range temperature measurements in medical thermography. Its compact size makes it suitable for integration into customizable systems. The sensor has a resolution of 160×120  pixels, which, although limited, is sufficient for studying the thermal variations of hemangiomas—small malformations with radii of a few centimeters. It delivers 8.7 frames per second, enabling users to view images in the system interface before capturing snapshots. This resolution and frame rate are compatible with real-time implementation of custom image processing algorithms, with results displayed directly on the system screen. This functionality provides valuable additional insights during patient consultations. After calibration, it can take thermal images and measure absolute temperature values within the visual scene.
• A Melexis MLX90614D IR thermometer to calibrate the thermal camera.
• A 5-inch HDMI LCD touch screen to display the images captured by the two image sensors and to control the user interface. The interface allows the simultaneous acquisition of visible and IR images.
• A standard 5 V battery to make the system autonomous and portable.

All the images were captured in a consultation room under indoor lighting provided by an light-emitting diode (LED) lamp, which exhibited an approximately Lambertian irradiance spectrum profile within the visible spectrum. The average room illuminance was 600 lux.

The thermal images are created within the device using an integrated Python routine,^5^^,^^6^ which calculates the temperature differences between the lesion center and the surrounding healthy skin. It then applies Otsu thresholding to ascertain the extent of the IH lesion in the IR thermal image. A false-color image representing diverse temperature values is presented on the device screen.

**Fig. 3:**
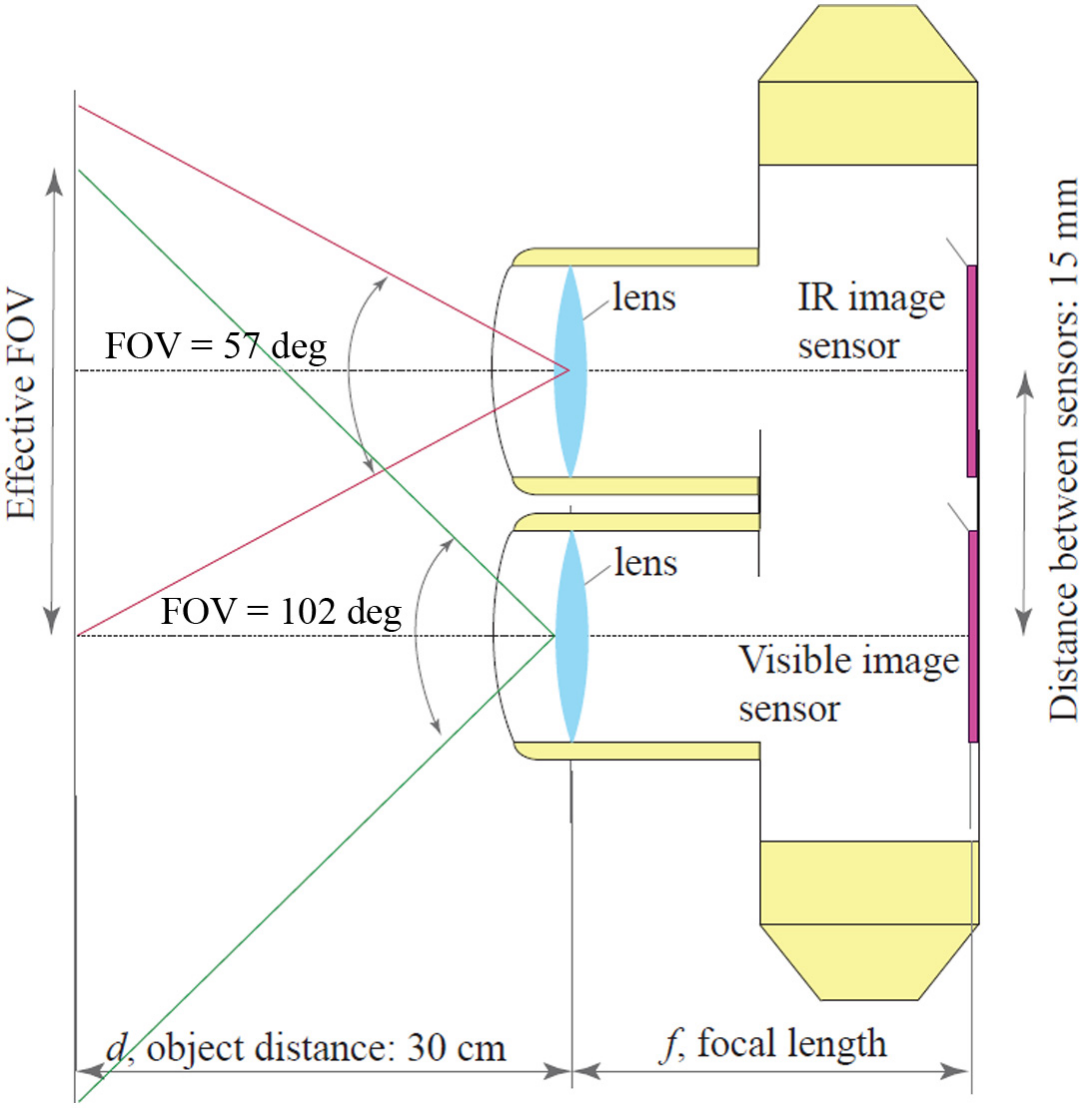
Optical configuration for the two sensors (visible and infrared) of the acquisition device.

Although no spacer was used, the optical setup remained consistent across all sessions. This arrangement required maintaining a fixed distance between the lesion and the system to ensure proper focus on the lesion. A basic schematic of the optical configuration for each sensor is provided in Fig. 3. The visible image sensor is equipped with a wide-angle lens with a 102 deg field of view (FOV). As the sensors are separated by only 15 mm, their images easily overlap when simultaneously capturing an object positioned 30 cm away. The FOV of the infrared sensor, despite its lower pixel resolution, is largely preserved. This avoids inefficient pixel usage in the IR sensor and ensures that lesions are effectively captured, provided they are properly aligned with the system.

The parameter d is the distance between the camera and the lesion that is fixed at 30 cm. The field of view is 102 deg in the visible sensor and 57 deg in the infrared one.

**Fig. 4:**
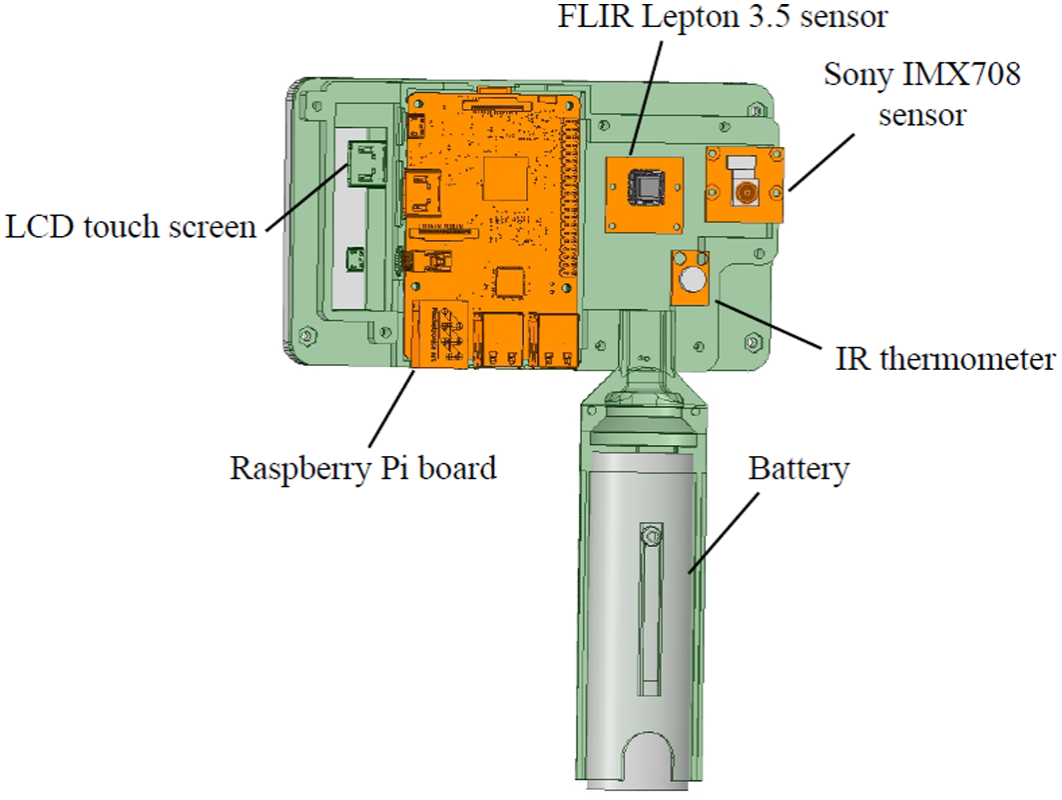
Primary image system acquisition peripherals.

In Fig. 4, we illustrate the arrangement of the primary image system acquisition peripherals.

### 2.2 Methods

**Fig. 5:**
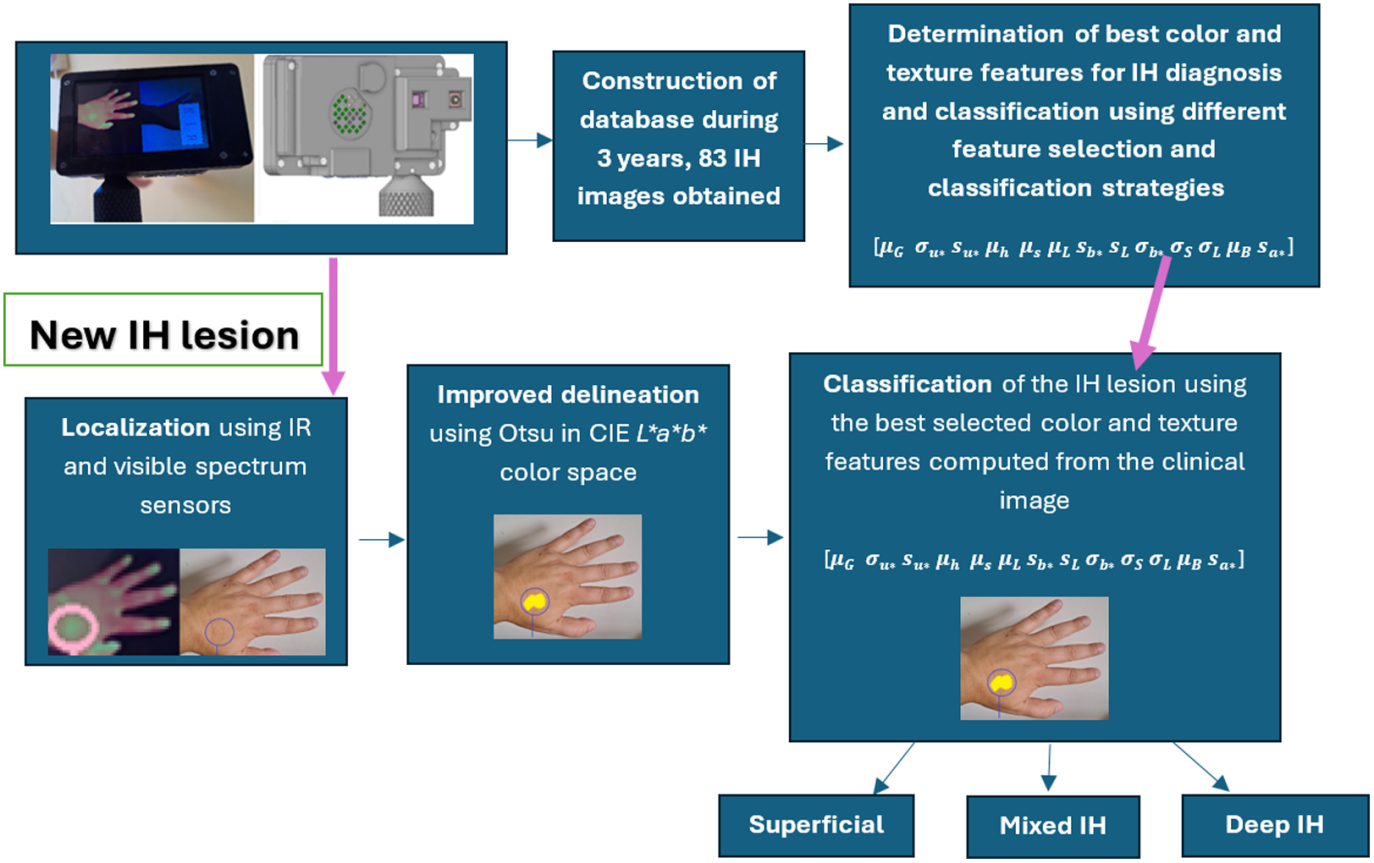
Workflow used in this study. The upper-left figure shows front and back views of the device developed by the authors.

The methodology proposed in this work consists of two steps, one to locate the IH lesion and determine its approximate extent, while a second to classify the lesion into one of the three subtypes (superficial, mixed, and deep). Precise lesion delineation is not essential for our current clinical goals. For the classification step, a section of the lesion is sufficient to calculate the necessary mathematical features. Figure 5 illustrates the workflow used in this study.

#### 2.2.1 Localization and extent

In clinical practice, IH lesions are localized through visual inspection, and in challenging cases, techniques such as Doppler ultrasound or MRI may be employed to accurately identify the lesions or differentiate them from other vascular anomalies. Thermal imaging assists dermatologists in detecting and categorizing lesions as IH, utilizing elevated temperature readings from the IR sensor within the device.^5^^–^^10^ Superficial IH lesions are typically easily discernible through visual inspection of the clinical image. Conversely, there might be deep or mixed IH lesions undetectable via the clinical image alone, but identifiable due to the heightened temperature values from the IR sensor. As previously shown by the authors,^6^^,^^30^ temperature information can be used to classify vascular lesions into fast-flow lesions (including IH and arteriovenous malformations) and slow-flow lesions [including malformations such as venous or lymphatic capillary abnormalities, such as port wine stains (PWS)]. Furthermore, they showed that temperature can also be used to determine the extent and surface area of IH lesions.^5^

**Fig. 6:**
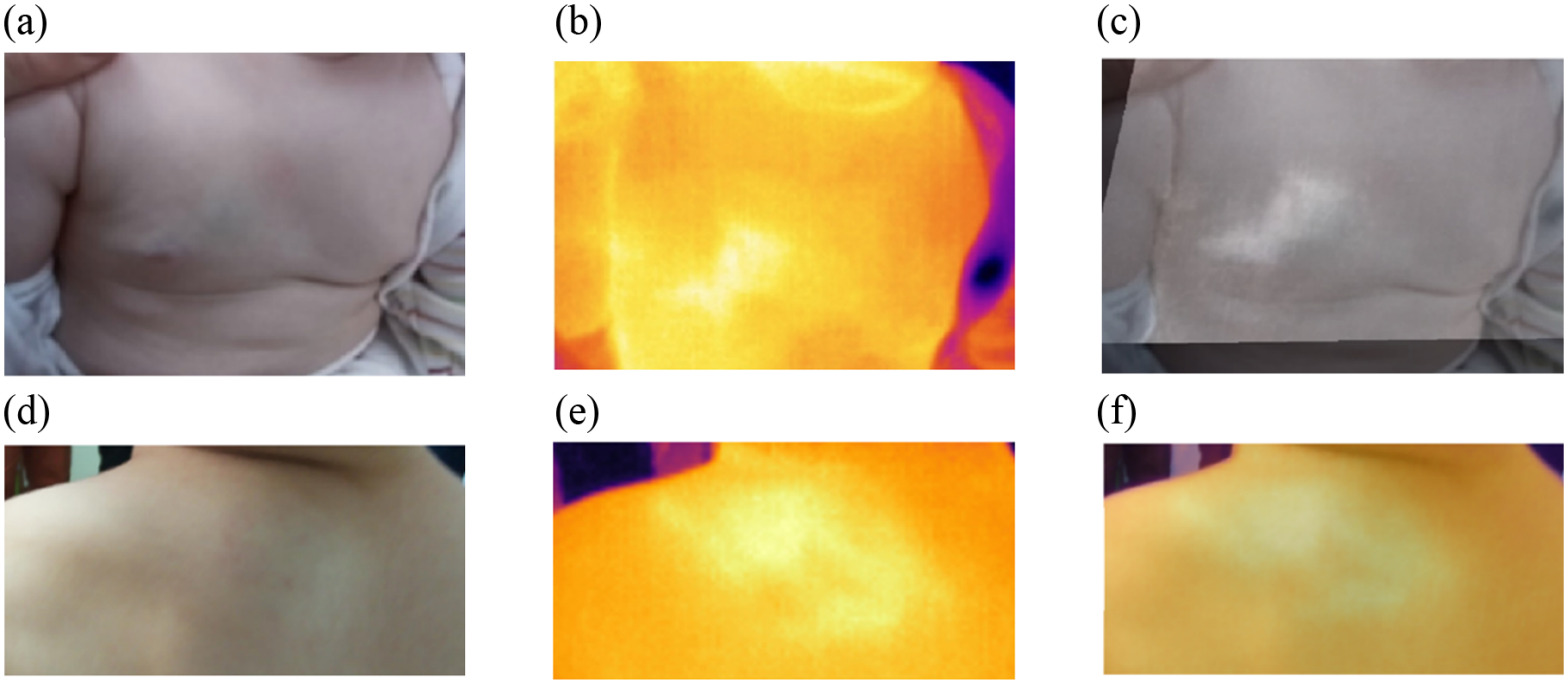
Panels (a) and (d) show clinical images of deep IH lesions. Panels (b) and (e) display the thermal IR image of the deep IH lesion, and panels (c) and (f) show the images obtained after registering the clinical and IR images.

The importance of this stage is illustrated through the clinical and thermal images of two deep IH lesions presented in Figs. 6(a) and (d). One can observe the challenge of distinguishing the lesion location and extent through visual inspection alone. The thermal images shown in Figs. 6(b) and 6(e) display higher temperatures within the lesion, aiding in precisely identifying their locations and extents. A registration process of both the thermal and clinical images is carried out. In this approach, the registration method is based on the improved curvature scale space algorithm as proposed by Li et al.,^31^ whose code is available online. Figs. 6(c) and 6(f) showcase the outcomes of the registration of the two images, providing a clearer depiction of the lesions’ locations and extents.

Once the lesion has been located, the extent of the lesion can be determined using the registered image converted to *Commission Internationale de l’Éclairage* (CIE) $L^{*}a^{*}b^{*}$ color space. More specifically, the lesion is delineated using an enhanced Otsu thresholding algorithm previously implemented by the authors.^32^ In this enhanced thresholding, the procedure is as follows:

1. First, the dermatologist selects a small cutoff or rectangle inside the lesion part with the mouse.
2. The average color of the pixels belonging within this selected area is calculated in CIE $L^{*}a^{*}b^{*}$ color space ($\overset{¯}{L^{*}}\overset{¯}{a^{*}}\overset{¯}{b^{*}}$).
3. A distance image is built where each pixel represents the difference between its color and the average color determined in step 2
  $$d(i,j)=\sqrt{(L^{*}(i,j)-\overset{¯}{L^{*}}{)}^{2}+(a^{*}(i,j)-\overset{¯}{a^{*}}{)}^{2}+(b^{*}(i,j)-\overset{¯}{b^{*}}{)}^{2}}$$
  In this sense, the area with color similar to the lesion will always be the darkest and will correspond to the leftmost peak in the histogram.
4. Subsequently, the algorithm incorporates some processing steps to find relevant peaks in the histogram, aiming to isolate the most significant left peak, which should correspond to the pixels belonging to the lesion.

This procedure is repeated for every image, obtaining for each one the detected hemangioma lesion. Subsequently, this segmented hemangioma is classified into its depth, as explained in Sec. 2.2.2.

#### 2.2.2 Depth estimation

After locating the lesion, the next step is to classify the IH lesion. To the best of our knowledge, no prior studies have concentrated on classifying hemangioma types. All published research has focused instead on segmenting infantile hemangioma lesions or classifying skin lesions into hemangioma and other lesion types.^11^^–^^20^ In the absence of comparative work, the established methodology has been to evaluate the various selection and classification methods available in the literature and to test the effectiveness of color and texture features of proven usefulness to assist in the diagnosis of other skin lesions and to examine their correlation with the diagnostic criteria for other skin lesions.

Color and depth are the descriptive features clinicians use for diagnosis and classification:^1^^–^^4^ superficial IHs typically present as bright red with a raised and uneven surface; deep IHs, being subcutaneous, are challenging to locate and often manifest as a subtle bluish purple swelling with a smooth surface; mixed IH lesions exhibit features of both superficial and deep hemangiomas. Identifying and calculating the mathematical color and texture features closely tied to those visual elements of color and depth is crucial for automatic diagnosis and would offer clinicians an explainable *in situ* assessment.

The classification stage involves two sequential tasks: feature extraction and image classification.

##### Feature extraction and selection

The methodology followed to determine the features to be introduced into the classifiers was to search for mathematical measures that correlated with the dermatologist’s description of the visual appearance of the skin lesion, i.e., the visual features that help the dermatologist to make a diagnosis. The clinical features described by the dermatologist were largely consistent with those used to diagnose other skin lesions such as burns (erythema, redness, swelling, and roughness). In a previous research by the authors,^33^^,^^34^ they conducted a psychovisual experiment and applied multidimensional scaling analysis to identify color-mathematical metrics that correlated with visual features of importance to clinicians. Thus, those mathematical features^32^ are the starting point in our experiments. In other words, the aim of this study is to test the usefulness of first-order statistics of different color measurements in distinguishing hemangioma subtypes.

The features selected in this paper are derived from the primary components of each color space ($R,G,B,L^{*},a^{*},b^{*},u^{*}$, and $v^{*}$), but they differ in essence and significance. The chromatic information in $L^{*}a^{*}b^{*}$ and $L^{*}u^{*}v^{*}$ color spaces is codified in a*b* and $u^{*}v^{*}$ components, respectively. In RGB color spaces, chromatic and achromatic information are codified mixed in the three components (R,G, and B). In addition, as $L^{*}a^{*}b^{*}$ and $L^{*}u^{*}v^{*}$ are uniform color spaces, color perceptual differences are correlated with Euclidean distances among pixels, which may benefit the learning of the classifier. The $L^{*}u^{*}v^{*}$ and $L^{*}a^{*}b^{*}$ color spaces were both standardized by the CIE in 1976 as perceptually uniform color spaces. At that time, there were no clear advantages between the two, so both were adopted. $L^{*}$ component is common to $L^{*}a^{*}b^{*}$ and $L^{*}u^{*}v^{*}$. But features extracted from ($u^{*},v^{*}$) and (a*,b*) components are likely to be highly correlated. However, as several feature selection (FS) methods have been implemented, the authors have delegated the selection of the most discriminant features to these methods.

To get the color information, we calculate the average values of the segmented IH lesion of the following color components:

– RGB values ($\mu_{R},\;\mu_{G},\;\mu_{B}$)
– Chromatic components: $a^{*},b^{*},u^{*}$, and $v^{*}$ ($\mu_{a*},\mu_{b*},\mu_{u*},\mu_{v*}$)
– Achromatic component: $L^{*}$ ($\mu_{L}$)
– Hue component ($\mu_{h}$)
– Saturation component (μs)

To get the texture information such as heterogeneity, softness, and rugosity, we calculate some metrics based on first-order statistics, that is, metrics derived from the histogram.^39^ They are as follows:

– Standard deviations of the components: $L^{*},a^{*},b^{*},u^{*},v^{*}$, hue, and saturation ($\sigma_{L},\sigma_{a*},\sigma_{b*},\sigma_{u*},\;\sigma_{v*}$, $\sigma_{h},\sigma_{s}$)
– Skewness of $L^{*},a^{*},b^{*},u^{*},v^{*}$ components $(s_{L},s_{a*},s_{b*}$, $s_{u*},s_{v*}$)
– Kurtosis of $L^{*},a^{*},b^{*},u^{*},v^{*}$ components $(k_{L},k_{a*},k_{b*}$, $k_{u*},k_{v*}$)

In total, the number of descriptors is 28. To reduce the large number of features and optimize subsequent classification, an FS step was conducted. In 2019, Venkatesh et al.^35^ published a review of various FS methods aimed at reducing feature size. For this study, six different selection techniques were chosen, each technique providing a ranked set of features from the most to the least discriminative ones. The selected techniques are outlined as follows:

• Univariate feature ranking using individual chi-square tests (will be referred to as chi-square). This method ranks the features using the p-values of the chi-square test statistics.
• Minimum redundancy maximum relevance algorithm (mRMR).^36^ In this case, mutual information is used to rank the features.
• Laplacian score for feature selection. This unsupervised learning method uses Laplacian scores^37^ for feature ranking.
• Feature ranking using the ReliefF and RReliefF algorithms^38^ with k-nearest neighbors (kNN) (referred to as *Relief* in Tables 3 and 4).
• Feature selection using neighborhood component analysis.^39^
• Sequential forward feature selection or sequential backward feature selection (SBFS). Our approach employs *N* support vector machine (SVM) models, where *N* represents the number of classes considered.

Each feature selection technique yielded a ranked list of features. To obtain the optimum set of descriptors from these lists, we sequentially pass each set of rank-ordered features to each classifier, adding a new feature in each iteration. The optimal feature set is the one that obtains the lowest classification error.

##### Classification

Evaluation of the classification performance for each ranked feature set was conducted using several classification schemes. In a study by Ponnusamy et al.,^40^ a literature review was performed on diverse strategies for image classification. Some of these methods were applied in our study, including

• Linear discriminant analysis (referred to as LDA),
• kNN,
• SVM.
• Multilayer perceptron (MLP) with sigmoid activation function in the output layer. The MLP classifier comprises a hidden layer with 67 nodes and employs the Adam optimizer without a learning rate scheduler and categorical cross-entropy as the loss function. The number of epochs was set to 30. The minibatch size was 16, and the learning rate was set to 0.001. The optimizer hyperparameters $\beta_{1}$ and $\beta_{2}$ were set to 0.9 and 0.999, respectively. Epsilon was set to $10^{-8}$.

To select the best MLP configuration, we tested various configurations and parameters and found that the model described above gave the best results during the validation stage. Our validation experiments included different optimizers such as Adam and stochastic gradient descent with momentum, along with various learning rates, minibatch sizes, and unit counts. Different experiments were conducted for the number of units in the hidden layer, varying from 30 to 200. Initially, the increments were set at 20 units, but as the results approached optimality in the accuracy value, the increments were reduced to 1. The optimal number of units was 67 (highest validation accuracy).

Given the limited availability of images, predominantly from the mixed and deep IH classes, we did not explore more complex classifiers such as deep neural networks.

## 3 Results

### 3.1 Localization and Extent

Table 1 shows the number of images correctly identified by a dermatologist with and without the acquisition device. The table provides the number of images in which the expert was able to correctly localize the lesion and its extent by visual inspection alone or with the aid of the handheld IR camera. This evaluation was performed with the naked eye of the expert, who judged whether he had underestimated the extent of the lesion before observing it with the IR camera. The fact that the same observer subsequently determines the extent of the lesion without and with IR information could lead to bias. However, it could also aid in a fair assessment of IR improvement. Although superficial and mixed subtypes were easily visible through visual inspection, some mixed IH lesions with depth components posed challenges for delimitation (specifically, 2 out of 27). In addition, the IR sensor was critical in determining the extent of IH lesions, as the expert judged that he had underestimated the extent of 4 out of 12 deep lesions using only visual inspection or the visible sensor.

**Table 1:** Localization experimental results with and without the IR sensor performed by a dermatologist during consultation.

Localization and extent	Number of images	Correctly located visually	Correctly located with IR sensor	Extent correctly determined visually	Extent correctly determined with IR sensor
**Superficial**	44	**44 (100%)**	**44 (100%)**	**44 (100%)**	**44 (100%)**
Mixed	27	27 (100%)	**27 (100%)**	25 (92,6%)	**27 (100%)**
Deep	12	10 (83,3%)	**12 (100%)**	8 (66,7%)	**12 (100%)**

The best results are highlighted in bold.

**Fig. 7:**
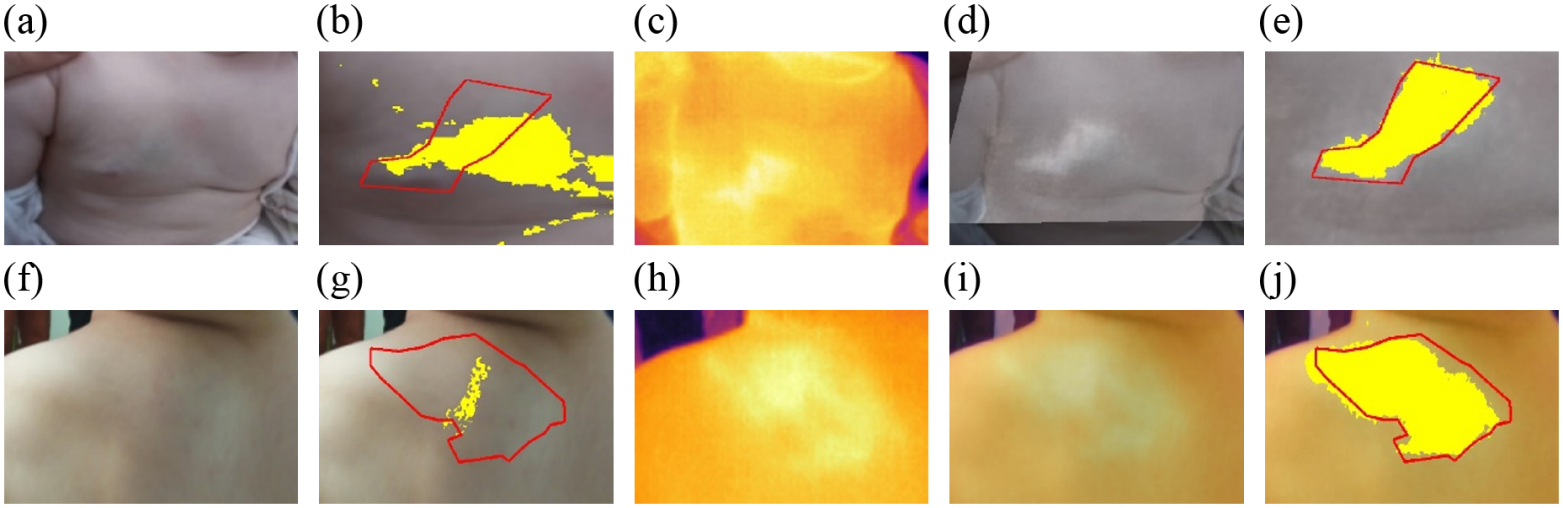
Segmentation results for deep IH lesions. Panels (a) and (f) show clinical images of deep IH lesions. Panels (b) and (g) show the automatic segmentation of both deep IH lesions (in yellow). The red line represents the ground truth segmentation performed by a clinical expert. Panels (c) and (h) display the thermal IR image of the deep IH lesion, and panels (d) and (i) show the image obtained after registering the clinical and IR images. Panels (e) and (j) show the segmentation results using the registered images.

Figure 7 illustrates the improvement made by adding the IR spectral band and the registration step to the method for determining the extent of the lesion in deep IH lesions. As can be observed in Figs. 7(a) and 7(f), deep IHs are not easily seen in visible images. Therefore, IR images can significantly improve the quality of the segmentation.

**Fig. 8:**
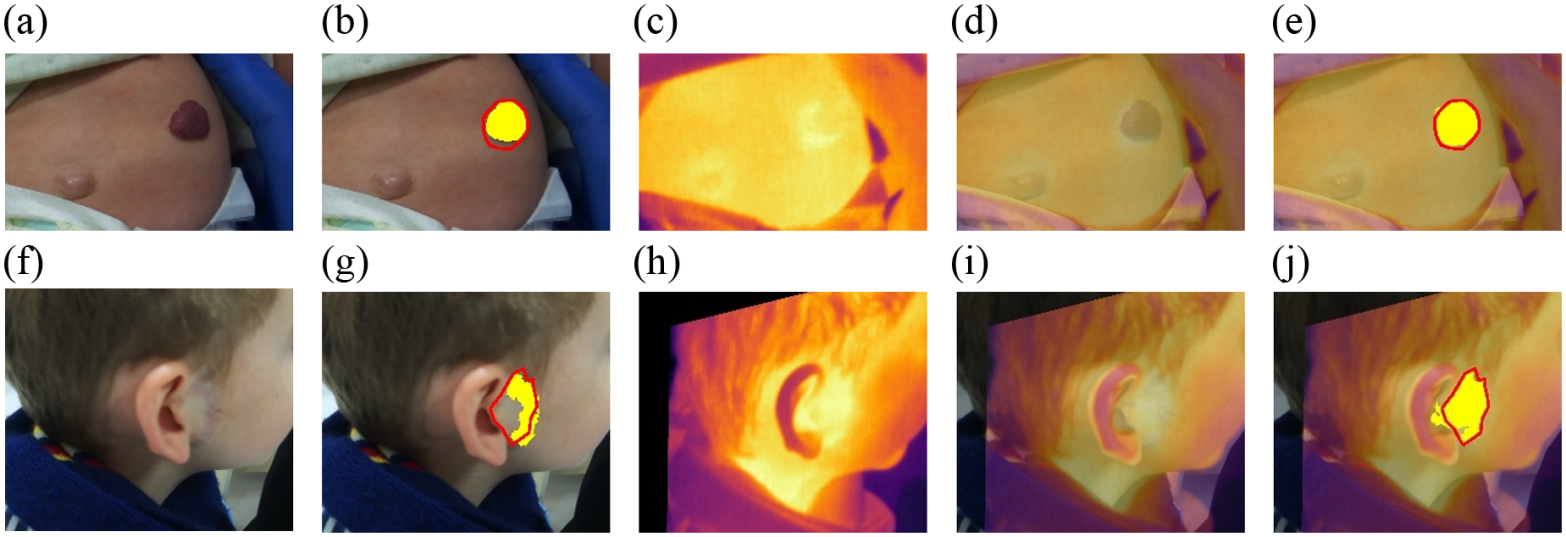
Segmentation results for IH lesions with superficial components. Panel (a) shows the clinical image of a superficial IH. Panel (f) shows the clinical images of a mixed IH. Panels (b) and (g) show the automatic segmentation of both IH lesions (in yellow). The red line represents the ground truth segmentation performed by a clinical expert. Panels (c) and (h) display the thermal IR image of the IH lesions, and panels (d) and (i) show the images obtained after registering the clinical and IR images. Panels (e) and (j) show the segmentation results using the registered images.

Figure 8 illustrates the segmentation results for two IHs with superficial components: a superficial IH [Fig. 8(a)] and a mixed IH [Fig. 8(f)]. Unlike the case of deep hemangioma, the area of the superficial component can be easily distinguished from healthy skin in the clinical image. Therefore, IR imaging hardly improves the segmentation results of these superficial components. In this sense, the segmentation of the superficial IH in Fig. 8(a) is acceptable in both Figs. 8(b) and 8(e). Similarly, the superficial component in Fig. 8(f) is adequately segmented in Fig. 8(g). However, the segmentation without IR fails to segment the deep component, as shown in Fig. 8(g).

In Table 2, three performance metrics quantify these segmentation results. These performance parameters show that for the deep cases, the use of IR significantly improves the values of BF score (contour matching score), Dice, and Jaccard indices. However, for the superficial IH lesions, the improvement, although important, is not as significant as for the deep cases.

**Table 2:** Segmentation performance metrics for deep, superficial, and mixed IHs.

	Contour matching score (BF score)	Dice index	Jaccard index
Clinical image without IR deep lesion [Fig. 7(b)]	0.0812	0.367	0.225
Registered image with IR deep lesion [Fig. 7(e)]	**0.356**	**0.883**	**0.791**
Clinical image without IR deep lesion [Fig. 7(g)]	0.041	0.147	0.079
Registered image with IR deep lesion [Fig. 7(j)]	**0.257**	**0.871**	**0.771**
Clinical image without IR superficial lesion [Fig. 8(b)]	0.904	0.846	0.733
Clinical image with IR superficial lesion [Fig. 8(e)]	**1.000**	**0.941**	**0.889**
Clinical image without IR mixed lesion [Fig. 8(g)]	0.7502	0.608	0.437
Clinical image with IR mixed lesion [Fig. 8(j)]	**0.915**	**0.878**	**0.783**

### 3.2 Feature Selection

For all feature selection and classification techniques mentioned in section feature extraction and selection, k-fold cross-validation was conducted with k=10. However, for the MLP classifier, 15% of the training dataset is reserved for validation to fix the hyperparameters. The most relevant features within each ranked subset and the optimal classifier were chosen according to two different criteria: F-score and accuracy maximization criteria.

Two distinct classifications were carried out: (1) categorization into three classes of IH—superficial, mixed, and deep; and (2) classification into two classes: superficial and mixed–deep. In the latter, the deep and mixed classes were merged due to the limited availability of deep IH images. This two-class classification was useful to clinicians as both deep and mixed lesions require similar medical management.

**Fig. 9:**
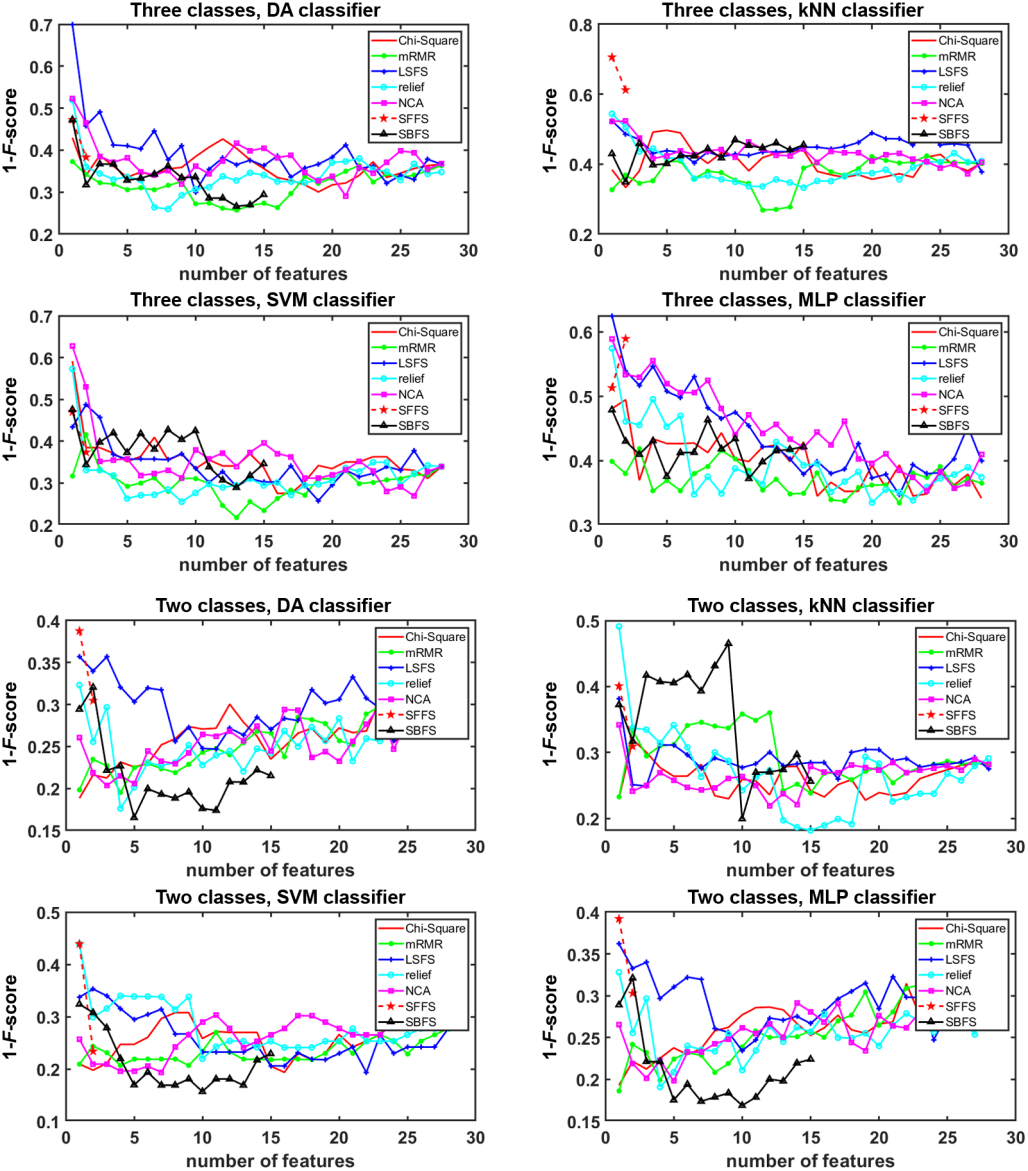
Classification error (1-F-score) versus the number of features incorporated from each feature selection method for the various classification techniques for the three- and two-class scenarios.

Figure 9 shows the classification error (1-F-score) on the vertical axis for the test set, plotted against the number of features used from each selection method for different classification techniques on the horizontal axis. Each point on the horizontal axis represents the number of features added to the classification stage. The positions giving the lowest error indicate the optimal number of features. For the three-class classification, the best selection method, as shown in Fig. 9, is mRMR for the four different classifiers (LDA, kNN, SVM, and MLP). For the two-class classification, the best selection methods are SBFS and Relief. Tables 3 and 4 show the classification results for these best cases, which will be explained in Sec. 3.3.

**Table 3:** Classification experimental results using the different classifiers and set of features, when the criterion to optimize the number of features is accuracy.

Classifier three classes	Feature selection technique	Final set of features	Accuracy	Sensitivity	Specificity
LDA	mRMR	[$\mu_{G}\sigma_{u*}s_{u*}\mu_{h}\mu_{s}\mu_{L}s_{b*}s_{L}\sigma_{b*}\sigma_{S}\sigma_{L}\mu_{B}s_{a*}$]	0.7572	0.8948	0.7313
**SVM**			0.7831	0.8564	0.7226
MLP			0.7527	0.7517	0.8558
kNN		[$\mu_{G}\sigma_{u*}s_{u*}\mu_{h}\mu_{s}\mu_{L}s_{b*}s_{L}\sigma_{b*}\sigma_{S}\sigma_{L}\mu_{B}s_{a*}\mu_{b*}]$	0.7253	0.8380	0.6024
Classifier two classes		Final set of features	Accuracy	Sensitivity	Specificity
LDA	**SBFS**	[$\mu_{h}\sigma_{L}\mu_{b*}\sigma_{a*}\sigma_{b*}$]	0.8313	0.8897	0.8937
**SVM**		[$\mu_{h}\sigma_{L}\mu_{b*}\sigma_{a*}\sigma_{b*}k_{L}s_{a*}k_{a*}s_{b*}\mu_{u*}$]	0.8434	0.8569	0.8616
MLP	Relief	$[\mu_{h}s_{L}\sigma_{S}\sigma_{u*}k_{L}\sigma_{a*}s_{b*}k_{v}k_{b*}\mu_{G}\sigma_{b*}$]	0.8352	0.8082	0.8518
kNN		$[\mu_{h}s_{L}\sigma_{S}\sigma_{u*}k_{L}\sigma_{a*}s_{b*}k_{v}k_{b*}\mu_{G}\sigma_{b*}s_{u*}\mu_{a*}\sigma_{h}\mu_{L}k_{u*}$]	0.8253	0.8925	0.8559

The best results are highlighted in bold.

**Table 4:** Classification experimental results using the different classifiers and a set of features when the optimizer criterion is *F*-score.

Classifier three classes	Feature selection technique	Final set of features	*F*-score	Sensitivity	Specificity
LDA	mRMR	[$\mu_{G}\sigma_{u*}s_{u*}\mu_{h}\mu_{s}\mu_{L}s_{b*}s_{L}\sigma_{b*}\sigma_{S}\sigma_{L}\mu_{B}s_{a*}$]	0.7436	0.7705	0.8627
SVM			0.7826	0.7803	0.8720
MLP			0.683	0.6755	0.8332
kNN		[$\mu_{G}\sigma_{u*}s_{u*}\mu_{h}\mu_{s}\mu_{L}s_{b*}s_{L}\sigma_{b*}\sigma_{S}\sigma_{L}\mu_{B}s_{a*}\mu_{b*}]$	0.7313	0.7382	0.8312
Classifier two classes		Final set of features	*F*-score	Sensitivity	Specificity
LDA	SBFS	[$\mu_{h}\sigma_{L}\mu_{b*}\sigma_{a*}\sigma_{b*}$]	0.835	0.8335	0.8335
SVM		[$\mu_{h}\sigma_{L}\mu_{b*}\sigma_{a*}\sigma_{b*}k_{L}s_{a*}k_{a*}s_{b*}\mu_{u*}$]	0.8433	0.845	0.845
MLP	Relief	$[\mu_{h}s_{L}\sigma_{S}\sigma_{u*}k_{L}\sigma_{a*}s_{b*}k_{v}k_{b*}\mu_{G}\sigma_{b*}$]	0.8313	0.8325	0.8325
kNN		$[\mu_{h}s_{L}\sigma_{S}\sigma_{u*}k_{L}\sigma_{a*}s_{b*}k_{v}k_{b*}\mu_{G}\sigma_{b*}s_{u*}\mu_{a*}\sigma_{h}\mu_{L}k_{u*}$]	0.8183	0.8164	0.8164

The best results are highlighted in bold.

### 3.3 Classification

Tables 3 and 4 display the optimal feature set leading to the highest accuracy and to the highest *F*-score in classification for each classifier, respectively. It also shows the feature selection techniques producing the top-ranked sets. Sensitivity and specificity metrics are provided as well. Both accuracy and *F*-score were used as stopping criteria to better analyze the imbalanced nature of the dataset. Accuracy and *F*-score consider false positives and false negatives, but *F*-score is considered a better metric when dealing with imbalanced datasets because it takes into account the type of errors and not just the number of predictions that were incorrect. Results in Tables 3 and 4 show that the accuracy maximization criterion favors high sensitivity, whereas the *F*-score criterion favors high specificity. In this sense, the best sensitivity (0.8948) for three-class classification is obtained with LDA and mRMR as feature selectors and accuracy as optimization criterion, whereas the best specificity is obtained with an SVM as classifier, mRMR as feature selector, and *F*-score as optimization criterion. On the other hand, the best configuration for binary classification is SVM as classifier, with SBFS as selector, and accuracy as criterion, which achieves a good balance between sensitivity and specificity.

Another remark is that the selected features in the three-class classifications include the third and fourth moment parameters, whereas only five features obtained from the mean and variance of different color metrics are sufficient to obtain a good performance in the binary classification. Specifically, the best specificity (0.8937) with almost the best sensitivity (0.8897) is obtained when the inputs to an LDA classifier are [$\mu_{h}\sigma_{L}\mu_{b*}\sigma_{a*}\sigma_{b*}$].

Given the small overall dataset size, to validate the robustness of the metrics in Tables 3 and 4, a leave-one-out procedure was performed to get the best performance configuration. For the three-class classification and *F*-score criterion, mRMR as feature selector and SVM as classifier, the final set of features was the same as the one obtained in Table 4, i.e., [$\mu_{G}\sigma_{u*}s_{u*}\mu_{h}\mu_{s}\mu_{L}s_{b*}s_{L}\sigma_{b*}\sigma_{S}\sigma_{L}\mu_{B}s_{a*}$]. The sensitivity was 0.7858, and the specificity was 0.8865 for a *F*-score equal to 0.794, very similar values to those obtained in the *k*-fold cross-validation.

**Fig. 10:**
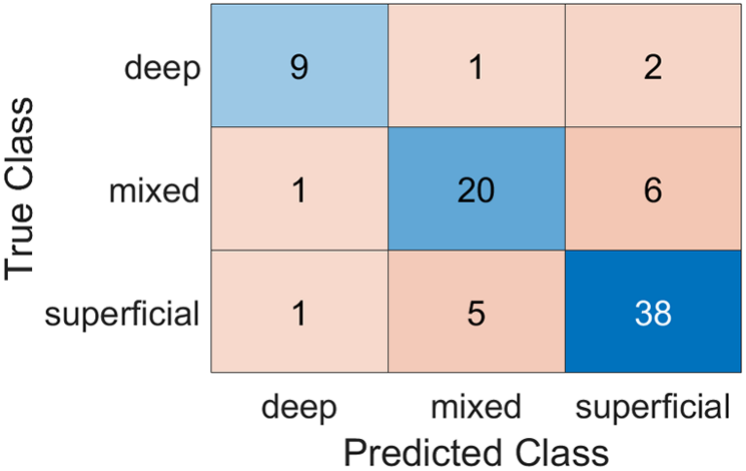
Confusion matrix for the three-class classification.

Finally, the confusion matrix is shown in Fig. 10. This matrix emphasizes that most errors are between superficial and mixed lesions. There is only one error between deep and mixed lesions and vice versa; and only three errors between deep and superficial IH or vice versa.

In the leave-one-out validation for binary classification, the best results were also obtained with an SVM classifier and SBFS feature selection. The sensitivity was 0.8864 and the specificity was 0.8462 for a *F*-score equal to 0.8764, in the order of those obtained in the *k*-fold cross-validation. The set of input features for these results was $\left[\mu_{h}\sigma_{L}\mu_{b*}\sigma_{a*}\sigma_{b*}\right]$. Interestingly, as happens in the *k*-fold cross-validation optimizing accuracy, only features obtained from the mean and variance of different color metrics are sufficient to obtain a good performance in the binary classification. This result aligns with the established perceptual distinctions among hemangioma subtypes. The identification of these subtypes is predominantly determined by the lesion’s mean color and its uniformity across the lesion.

## 4 Discussion

The challenge of segmenting IHs and detecting IHs among other vascular lesions has been addressed in various attempts in the literature.^21^ However, to the best of our knowledge, no attempt has been published to segment deep vascular lesions that are barely visible in clinical images. In this paper, we have addressed the challenge of localizing and estimating the extent of deep lesions by combining the clinical and IR images, showing the improvement gained with the additional help of IR information.

To the best of our knowledge, there are no previously published works that have addressed the problem of classifying IHs into their three subtypes: superficial, mixed, and deep. This is a key classification to decide the appropriate treatment and to evaluate the prognosis.^3^

To solve this problem, we addressed two challenges. We had to identify mathematical characteristics that correlate with clinical descriptions or clinical signs that lead the dermatologist to a certain diagnosis. As previously published, hue and colorfulness measured in uniform color spaces correlate well with dermatologists’ color descriptions of skin lesions such as bright red, bluish cast, or normal skin color,^33^^,^^34^^,^^41^ which are distinctive color features of IH subtypes.^3^ Likewise, statistical parameters derived from the histogram can measure typical skin texture descriptions such as scaliness or brightness.^33^^,^^34^^,^^41^ The classification results presented in this paper confirm the validity of these features to analyze IH subtypes. In this sense, the second important contribution of this paper is the identification of mathematical features capable of clinically describing IHs, with the added advantage of the explainability of the categorization. This set of features is [$\mu_{G}\sigma_{u*}s_{u*}\mu_{h}\mu_{s}\mu_{L}s_{b*}s_{L}\sigma_{b*}\sigma_{S}\sigma_{L}\mu_{B}s_{a*}$]. It includes the mean of the lightness, saturation, and hue values as well as G and B color components, which are closely related to color and brightness information, and the standard deviation of several color components derived from both chrominance and luminance information, which measures their variability along the lesion. Intuitively, this information can be used to differentiate between superficial and deep IH lesions, which typically appear as red and bluish purple, respectively. Finally, the inclusion of the skewness of the two chrominance components indicates the importance of the symmetry of the color distribution for the classification of IH depth as the asymmetry, especially in the luminance component, is typically associated with shadows and highlights, and thus related to the possible appearance of rugosity or moisture. The calculation and usefulness of the selected features are discussed in detail in the color reference literature.^41^ The selection of these features is based on previous research by the authors^33^^,^^34^ in which a psychovisual experiment identified color-mathematical metrics that correlated with visual features of importance to clinicians, such as roughness and erythema, which are highly prevalent in skin lesions. This study has validated the hypothesis that these features are able to classify hemangioma subtypes. Nevertheless, interesting future research could be to test second-order statistics-based features to see if a further improvement in classification results could be achieved. For example, it would be interesting to compute texture parameters extracted from co-occurrence matrices, which could provide information about local spatial relationships.

As there have been no previous attempts to classify IHs into their subtypes, our second challenge was to identify the feature selection method and the more appropriate classifier to approach this task. Therefore, several machine learning methods were tested. According to Tables 3 and 4, the mRMR algorithm excels in three-class classification and the SBFS algorithm in two-class classification, with both accuracy and *F*-score as stopping criteria and with both *k*-fold cross-validation and leave-one-out validation methods.

It is essential to emphasize that the available image pool is currently limited and that there is an absence of public databases. Existing literature on infantile hemangioma lesions faces the same constraint.^5^^–^^20^^,^^29^ Besides, prior research has not classified IH lesions. Existing studies have solely focused on delineating visually identifiable superficial and mixed types (overlooking their potentially subcutaneous and nonvisible deep components). Consequently, it is not possible to compare this with other strategies or prior work at this time.

As stated above, in this paper, the validity of the color and texture features chosen for the distinction of the three subtypes of hemangiomas has been demonstrated. However, if we wonder about the ability of these features to distinguish among different vascular malformation types, as a possible future work, we will have to take into account the following facts:

1. PWS lesions may resemble superficial hemangiomas because they are flat pink capillary malformations, but they are slow-flow vascular malformations and, thus, are well differentiated from IH when high temperature is detected.^42^
2. Other fast-flow lesions besides hemangiomas are arteriovenous malformations. Being fast-flowing, they could be confused with hemangiomas on IR imaging, but they have a different clinical behavior, with a soft surface and a bluish component.^43^

An additional problem is the imbalanced nature of the database due to the uneven occurrence of the three hemangioma subtypes. Although the confusion matrix in Fig. 10 indicates that the classification is not particularly impacted by this imbalance, the implementation of cost-sensitive methods is necessary to address this issue effectively.^44^

## 5 Conclusion

The low-cost multisensor handheld device described in this study provides, for the first time, the integration of color, texture, and temperature data to enhance the visual diagnostic capabilities of physicians for immediate, accurate localization and classification of hemangioma lesions, all without the need for invasive or expensive methods that can prolong processes and increase healthcare costs. In fact, the device does two things: it helps clinical teams diagnose, localize, and assess the extent of hemangiomas in real time using thermal imaging, and it helps classify hemangiomas into one of three subtypes based on color and texture descriptors derived from clinical images.

It should be emphasized that, according to Mohamed and Rabie,^21^ current published studies focus primarily on the location and segmentation of IH lesions and the evaluation of treatment response. Thus, an important contribution of this paper is that it provides a method for classifying IHs into their subtypes.

In addition, some of these works have reported accurate results in IH segmentation.^11^^,^^13^^,^^17^ However, they have not reported the segmentation of deep IHs. In those IH lesions, the segmentation would probably not be so precise as these lesions have components below the skin surface that are not visible to the naked eye or traditional image analysis techniques. The inclusion of an LWIR sensor in our device, together with some Python routines, enables accurate localization of IH lesions, regardless of their subtypes.

Finally, the proposed approach to classify IHs into their subtypes is transparent in the sense that the dermatologists can know the visual features that motivated the AI classification as they are directly related to their way of diagnosing, unlike other deep learning methods proposed in the literature.^16^^–^^20^^,^^29^ This promotes an interpretable diagnosis and moves away from the opaque nature of deep learning models.

## Data Availability

The Python source code developed to control the system and process image data is available in the following public GitHub repository: https://github.com/juanle82/Biomedical_IR_Project.git The data underlying this paper will be shared at the reasonable request of the corresponding author.
